# The Persian version of the psychological flexibility in sport scale: a psychometric study

**DOI:** 10.1186/s40359-022-00962-x

**Published:** 2022-11-04

**Authors:** Farzaneh Badinlou, Rokhsareh Badami, Gustaf Reinebo, Markus Jansson-Fröjmark, Fahimeh Sepehri, Shiva Molaviniya, Tobias Lundgren

**Affiliations:** 1 Centre for Psychiatry Research, Department of Clinical Neuroscience, Karolinska Institutet & Stockholm Health Care Services, Region Stockholm, Stockholm, Sweden; 2 Department of Physical Education and Sports Sciences, Isfahan (Khorasgan) Branch, Islamic Azad University, Isfahan, Iran

**Keywords:** Athletes, Psychological flexibility, Psychometric properties, Reliability, Sport, Validity

## Abstract

**Background:**

There is a growing body of research suggesting that psychological flexibility (PF) is an important psychological construct related to psychological health and human performance. The Psychological Flexibility in Sport Scale (PFSS) is the first general scale to assess sport-related PF. So far, the PFSS has not yet been validated in other contexts than Sweden. Therefore, the current study sought to investigate a Persian version of the PFSS (P-PFSS) and extend the investigation of the psychometric properties of the PFSS in Iranian athletes.

**Methods:**

A total of 302 athletes from both team and individual sports (average age of 20.7 years, SD ± 7.5, 62.3% were female) were involved in the current study. Statistical analysis was performed on the data to test validity and reliability. The validity of the P-PFSS was tested through face and content validity, construct validity, criterion validity, and known-groups validity. The reliability of P-PFSS was verified through internal consistency and temporal stability of the scale.

**Results:**

Results revealed that validity of the P-PFSS was satisfactory. The instrument was determined to have strong face and content validity. With modifications, the confirmatory factor analysis confirmed the scale’s unidimensionality. The convergent validity of the P-PFSS was found to be acceptable (average variance extracted = 0.66) and satisfactory results were also found in the correlation matrix for the assessment of construct validity. The P-PFSS showed good criterion validity related to generic psychological flexibility and athletic-related variables. Also, the P-PFSS was able to differentiate PF between known groups. The P-PFSS was found to be reliable, with good internal consistency (Cronbach’s alpha = 0.92; composite reliability = 0.92) and temporal stability on retest (intraclass correlation coefficient = 0.95).

**Conclusions:**

Overall, the Persian version of the PFSS showed good psychometric qualities in Iranian athletes. The current study provides additional support for the PFSS and extends the context-specific utility for practitioners and researchers in assessing sport-related PF.

## Introduction

Athletes will encounter psychological barriers in their careers and struggle with thoughts, emotions and behavior problems that have a negative impact on both performance and well-being [1]. In order to understand athletes’ psychological performance difficulties and to target interventions that address these obstacles, it is important to identify, conceptualize, and measure psychological constructs central to the athletic endeavor.

Psychological flexibility (PF) is defined as the ability to be in continuous contact with the present moment, taking an accepting stance towards inner experiences while behaving in a values-based direction in your life [2]. PF is central to Acceptance and Commitment Therapy (ACT) [3], a behavioral change model shown to have positive effects in numerous areas of psychopathology and human functioning [4]. For little less than two decades ago, ACT and other cognitive and behavior therapies (CBT) focusing on acceptance, values, mindfulness or metacognitive processes [5], were adapted to athletes and the world of sports [6]. Mindfulness- and Acceptance-based (MA) methods are now widely used with athletes and performers to support and enhance effectiveness in sports. In a meta-analysis, MA methods were shown to have an influence on mindfulness measures, physiological, and psychological performance surrogates, and direct performance in precision sports [7]. While the application of MA interventions in sports rapidly increases, there is a growing need to develop and investigate measurements that target PF in relation to performance in order for researchers and practitioners to be able to measure both training processes and the effects of MA training.

A psychologically flexible athlete, as described in Lundgren et al. (2018), is being open toward aversive inner experiences (e.g., thoughts, emotions, memories, physiological responses) without trying to change the frequency or form of these events. The athlete’s actions are guided by her/his athletic and human values in practice, competition and in general life. Further, the athlete is aware and mindful about the unfolding events (internal and external) in order to be open and able to consciously pursuit an effective performance in a manner closely linked to personal values and meaning [8]. Up to date, there are two psychometrically investigated measures of PF adapted to athletic populations. The first is a sport-specific ice hockey PF measure; the Values, Acceptance and Mindfulness Scale for Ice Hockey (VAMS) [8]. The VAMS was evaluated in a Swedish sample of ice hockey players and constitutes of three factors central to PF (acceptance, mindfulness, and values) and showed an acceptable internal consistency (Cronbach’s α = 0.76). Also, the VAMS predicted objective ice hockey performance as measured by assists and team points. The second scale recently developed, is the Psychological Flexibility in Sport Scale (PFSS) [9]. The PFSS is a general sport PF measure, which enables the assessment of sport-related PF in a broad range of athletes. The PFSS was originally evaluated in a relatively small sample of Swedish athletes and was shown to constitute of one factor (seven items in total) and having satisfactory psychometric qualities. Since VAMS is a sport specific (ice hockey) measure of PF and the PFSS is a general sport measure, the PFSS was chosen as the basis for the adaptation described in this article due to the potential of its wide utilization.

The accessibility of psychometric scales through translation and cross-cultural adaptation is central to research and development in psychological sciences [10]. In addition, pervious study emphasizes cross-cultural differences in general PF [11]. A recent study was conducted in Iran and examined the psychometric properties of the Swedish PF scale in a clinical context. The results showed that the factor analysis of the Persian version did not produce the same structure as the Swedish version, which shed light on the relationship between culture and PF [12]. The findings of a recent review suggest that in some cases, context specific measures of PF are superior to general measures of PF regarding incremental validity and preferable regarding treatment sensitivity [13]. Although the PFSS is a context specific measure of PF in terms of being adapted to sports, it has not yet been evaluated outside a Swedish context. Translating and validating scales in multiple athlete populations is yet another step in making a measure context specific to improve its practical utility and gain further understanding of the psychometric qualities from updated and revised versions of the scale in populations not previously investigated. As a result of the expanding use of MA interventions worldwide [7, 14], it is important that the adaptations and translations of measures that are related to PF (and other constructs related to MA interventions) follow.

The translation and cultural adaption of the P-PFSS is also part of the general progress to strengthen the understanding for the role of PF in sports. In the original version and initial investigation of the PFSS, basic psychometric properties regarding validity and reliability (Cronbach’s alpha) were investigated [9]. However, further studies using the PFSS will need to be conducted to improve the understanding of PF in athletes and what measurement qualities the different versions of the instrument have and can be used for.

The purpose of the current study was therefore to translate, adapt, and psychometrically evaluate the PFSS for Persian speaking athletes in Iran. More specifically, this study aimed to evaluate the psychometric qualities of the PFSS regarding its (I) validity (face and content validity, construct validity, criterion validity, and known-groups validity), and (II) reliability (internal consistency and temporal stability) in a sample of Persian speaking athletes in Iran.

## Materials and methods

### Participants

The participants were recruited using a convenience sampling method through collaboration with the National Olympic Academy of Iran, sports Federations of Iran and sport clubs in Iran. The main inclusion criterion was that the participants are currently training and competing in their main sport. The sample size in the current study was estimated according to guidelines for sample size in psychometric properties research and the required sample size for factor analysis [15–18]. By considering a Cronbach’s alpha interval estimation between 0.8 and 0.85 and a significance level of 0.05, and a sufficient sample size to perform the factor analysis and an approximately 20% drop-out rate; a total of 380 participants were invited to take part in the study. The following exclusion criteria was used; (a) didn’t complete scales, or (b) had injuries during the past month which prevented participation in sports. The final sample consisted of 302 participants, aged between 15 and 42 years.

### Procedure

Athletes were recruited from team sports (54.4%); football (n = 63), volleyball (n = 61), basketball (n = 11), handball (n = 21), rugby (n = 5) and individual sports (45.6%); karate (n = 22), taekwondo (n = 20), tennis (n = 40), swimming (n = 14), canoeing (n = 13), wrestling (n = 2), gymnastics (n = 7), judo (n = 3), cycling (n = 2), athletics (n = 6), mountaineering (n = 2) and martial arts (n = 4). Data were collected through a web-survey using Google Forms. The Google Form link was distributed via internal email lists provided by the National Olympic Academy of Iran, sports Federations of Iran and general sport clubs.

The procedure was approved by the local Ethics Commission, a university board associated with the Islamic Azad university, Isfahan, Iran. Written informed consent was obtained from all included participants. For participants under 18 years of age, a written informed consent was also obtained from parents/guardians. All data collection procedures in the current paper follow the ethical standards of the Helsinki Declaration of 1964 and subsequent amendments [19].

## Measures

### Background questionnaire

The background questionnaire consisted of questions regarding age, gender, marital status, education, and self-rated economic status. In addition, the respondents were asked about age of onset of training for their main sport, type of sport, years of practicing the sport, years of competing and competition level (see Table 1).

### Athletic-related variables

Athletic-related variables consisted of three categories including preparation, competition, and recovery. A visual analogue scales (VAS) was used to measure these variables. The VAS is a widely used in the context of sport [20, 21] and it contains a range from 0 to 100 (0 = “extremely bad”; 100 = “extremely good”). Preparation; the athletes were asked to rate their average performance in preparing themselves before their recent match/competition, from 0 to 100 on the scale. Two preparation ratings were collected: one for physical preparation and one for mental preparation. Competition; the athletes were instructed to rate their performance during their recent match/competition from 0 to 100 on the scale. Recovery; the athletes were asked to rate how well they recovered themselves after their recent match/competition from 0 to 100 on the scale. Two recovery ratings were collected: one for physical recovery and one for mental recovery.

### Psychological health

The Depression, Anxiety, and Stress Scale (DASS-21) was used to measure psychological health in the current study. This scale was developed to measure depression, anxiety, and stress symptoms experienced during the past week. It is a 21-item scale and items are rated on a 4-point scale ranging from 0 (did not apply to me at all) to 3 (applied to me very much, or most of the time) [22]. The calculation of the scores acquired from each subscale was duplicated by two. Psychometric properties of the DASS-21 in an Iranian sample have been investigated previously. The results revealed that the Persian translation of DASS-21 has satisfactory psychometric properties [23]. The Cronbach’s alphas of the DASS-21 were α = 0.90 for depression, α = 0.83 for anxiety, and α = 0.91 for stress based on the current sample.

### Quality of life

The Satisfaction with Life Scale (SWLS) was used to measure quality of life in the present study. The SWLS is a 5-item scale measuring global life satisfaction. The items are rated on a 7-point scale, from 1 (strongly disagree) to 7 (strongly agree) [24]. The psychometric properties of the Persian version are acceptable [25]. The SWLS has been shown to display adequate test–retest reliability (Pearson’s r = 0.69), internal consistency (Cronbach’s α = 0.83), and concurrent and convergent validity [26]. The internal consistency of the SWLS was α = 0.83 based on the current sample.

### Generic psychological flexibility

The Acceptance and Action Questionnaire-II (AAQ-II) is a 7-item scale used to measure generic psychological flexibility in the current study. The items in the AAQ-II are rated on a 7-point Likert-type scale from 1 (never true) to 7 (always true). Higher scores on the AAQ-II are reflective of greater experiential avoidance and immobility, while lower scores reflect greater acceptance and action [27, 28]. The AAQ-II has retained good internal consistency (α = 0.85) and convergent and discriminant validity in an Iranian sample [29, 30]. The internal consistency of the AAQ-II was α = 0.88 in the current study.

### The psychological flexibility in sport scale (PFSS)

The PFSS was designed for measuring psychological flexibility in athletes. The PFSS was shown to be a valid unidimensional scale with acceptable psychometric properties in Swedish sample [9]. The scale consists of 7 items and each item is rated on a 7-point Likert-type scale, from 1 (never true) to 7 (always true), with higher scores indicating less psychological flexibility. Participants in the present study were asked to answer the questions of the PFSS considering the last month.

### Cross-cultural translation of the PFSS

The translation process consisted of the following steps according to the standards set by International Test Commission Guidelines for Test Adaptation [10]. In the first step, the PFSS was translated into Persian by two native Persian persons who were fluent in Swedish with a related background. Each person provided an independent translation of the items, instructions, and answer options. These two versions were compared and synthesized into the final version. In the second step, the Persian questionnaire was back-translated into Swedish by a professional Swedish translator who was also fluent in Persian. Then, the original Swedish and back-translated versions were compared by the first author, and no significant differences emerged.

### Psychometric analyses

Analyses were performed with SPSS version 27.0 and SPSS AMOS version 26.0. First, a descriptive analysis of the background variables is presented in Table 1. The minimum, maximum, means, and standard deviations of the psychological health, quality of life, and generic psychological flexibility scales are presented in Table 2. Then, the validity of the P-PFSS was estimated using face and content validity, construct validity, and criterion validity [31]. Moreover, known-groups validity of the P-PFSS was tested in the current study. The reliability of the P-PFSS was tested using internal consistency and temporal stability of the scale.

### Face and content validity

Face validity was tested by qualitative face validity. Thirteen Persian-speaking athletes (61.5% female, mean age = 25.1, *SD* = 2.3) were asked to complete the questionnaire and then interviewed in order to assess the participant’s interpretation of the items in the Persian version. Participants were also asked to rate each item in regards to how clear the meaning of the items to them on a 5-point Likert scale, from 1 = very clear to 5 = not clear at all. The content validity was assessed by expert evaluation. Four experts, including two psychologists (experts in ACT) and two faculty members (experts in physical education and sports sciences) were asked to review items and give feedback on the wording and scaling of the items. They were also asked to determine relevancy of each item on a four-point Likert scale from 1 (irrelevant) to 5 (completely relevant) [32]. Content validity index (CVI) was calculated for each item and the CVI average [33]. CVI greater than 0.80 is used as a recommended value [34]. Finally, proofreading was performed and the final version of the Persian scale was drafted. The scale was named the Persian version of the Psychological Flexibility in Sport Scale (P-PFSS) and distributed to the current study’s participants.

### Construct validity

The construct validity of the P-PFSS was evaluated through factorial validity and convergent validity. The factorial validity was assessed by conducting an exploratory factor analysis (EFA) and a confirmatory factor analysis (CFA). In EFA, Maximum Likelihood Extraction and Promax rotation method, assuming components to be correlated, were used in order to determine factor structure [35]. The factor loading ≥ 0.40 and accounting for > 10% of variance were considered as the requirements of satisfied extraction [36, 37]. Factorial validity was also evaluated by performing a confirmatory factor analysis to determine the fit of the statistical model with the data based on the results from the EFA. Two dimensions of fit statistics were considered to determine the model fit of the data: (a) absolute fit was measured using the chi-square goodness-of-fit index (p-value > 0.05), root mean-square error of approximation (RMSEA < 0.08), and standard root-mean-square residual (SRMR < 0.08) and (b) incremental fit was calculated using the comparative fit index (CFI ≥ 0.90) and the Tucker–Lewis Index (TLI ≥ 0.95) [38, 39]. Based on the final model, the convergent validity was calculated by the average variance extracted (AVE). Values of AVE ≥ 0.50 were considered acceptable [40, 41]. In addition, the construct validity was evaluated by calculating Pearson’s correlations between the P-PFSS and psychological health outcomes and quality of life in the current study.

### Criterion validity

The criterion validity of the P-PFSS was verified through concurrent validity and predictive validity. The concurrent validity of the P-PFSS was evaluated using correlation analysis between the P-PFSS and a well-established instrument for the same construct, the AAQ-II. The predictive validity was calculated using Pearson’s correlation coefficients to examine the association between the P-PFSS and the athletic-related variables. Our hypothesis is that a measure of PF should be able to predict how well athletes will perform professionally.

### Known-groups validity

The known-groups validity was determined by comparing P-PFSS total score depending on background variables by calculating Student’s t test and one-way analysis of variance (ANOVA). Post-hoc comparisons were performed using Tukey’s test.

### Reliability

The scale’s reliability was assessed by internal consistency and temporal stability of the P-PFSS. The internal consistency was evaluated using Cronbach’s alpha coefficients and the composite reliability (CR). A range of 0.70 to 0.95 was considered to be acceptable values for the Cronbach’s alpha coefficient [42, 43]. The CR ≥ 0.70 was considered adequate [44]. In addition, we evaluated the test-retest reliability of the P-PFSS using the Intraclass Correlation Coefficient (ICC) with a non-random partial convenient sample over a four-week period (n = 37). The ICC ≥ 0.70 was considered acceptable [44].

## Results

### Descriptive statistics for participant characteristics and measures

Demographic information about the participants is presented in Table 1. The sample included 302 athletes with an average aged of 20.76 years (SD = 7.5). The mean age of onset of training for the main sport, practicing the sport, and competing were 9.3 (SD ± 3.5), 7.3 (SD ± 5.7) and 6.06 (SD ± 5.11), respectively. In total, 37 participants were included in the test-retest analysis with an age range between 15 and 39 years old, (M = 25.55, SD = 6.4 years old). The majority of the participants in the test-retest sample were female (n = 27), had a university/ post graduate education level (n = 24), and played in team sports (n = 22).

**Table 1:** Background characteristics of the participants (N = 302)

Variable	n	%
Gender		
Female	188	62.3
Marital status		
Single	243	80.5
Married	38	12.6
In a relationship	21	7
Education		
High school	163	54
Diploma	52	17.2
Bachelor	55	18.2
Master and higher	32	10.6
Self-rated economic status		
Lower-middle and middle	188	62.3
Upper middle	114	37.7
Competition level		
Beginner	45	14.9
Club or university	66	21.9
Provincial	109	36.1
National	58	19.2
International	24	7.9
Sport type		
Individual	135	45.6
Team	161	54.4

Table 2 presents the minimum, maximum, means, and standard deviations of the psychological health, quality of life, and generic psychological flexibility scales.

**Table 2:** Descriptive statistics of the validity measures (N = 302)

Study scales	Min	Max	Mean	SD
Psychological health				
Depression	0	42	7.98	9.68
Anxiety	0	42	4.64	6.69
Stress	0	42	9.35	10.15
Quality of life				
Satisfaction with Life Scale	7	35	26.60	6.03
Generic psychological flexibility				
Acceptance and Action Questionnaire	7	49	14.49	7.89

### Face and content validity

The translated scale was used for the face validity estimation. A number of minor changes in the form of small text corrections were made for item 2 and item 7 to ensure adequacy of terminology and handle spelling mistakes. Subsequently, the P-PFSS scale was reviewed for the content validity by experts. The CVI of the items ranged from 0.83 to 1, and the scale’s CVI was 0.90. The CVI index of 0.80 and higher was considered acceptable. Based on the results, all seven items were included for the validation process.

### Construct validity

An EFA was conducted to explore the factor structure of the P-PFSS. The Kaiser-Meyer-Olkin test (0.91) and the Bartlett test of sphericity (χ^2^ (21) = 1548.35, p < 0.001) indicated the suitability of the data for factor analysis. The EFA results show the emergence of one factor explaining 69.65% of the total variance. All items had statistically significant loadings (0.76 − 0.90). The standardized factor loadings, means, standard deviations, Cronbach’s alphas, and intraclass correlation coefficients for the Persian version of PFSS are shown in Table 3.

**Table 3:** Standardized factor loadings, means, standard deviations, Cronbach’s alphas, and intraclass correlation coefficients for the P-PFSS (N = 302)

PFSS Items	Factor loadings	M	SD	α	ICC
1. My memories and experiences from previous failures have a negative impact on me when I am performing.	0.76	2.05	1.43	0.92	0.97
2. When competing I cannot control my nervousness, and my nervousness affects my performance negatively.	0.76	2.06	1.44	0.92	0.92
3. When I am competing my thoughts impair my performance.	0.90	2.29	1.59	0.90	0.85
4. When I am competing my feelings impair my performance.	0.88	2.09	1.42	0.90	0.92
5. It seems that most athletes can handle their feelings better than I do when they are competing.	0.84	2.21	1.58	0.91	0.93
6. Performance anxiety impairs my performance during competitions.	0.79	2.68	1.71	0.92	0.91
7. Worry makes my performance worse when I am competing.	0.87	2.48	1.67	0.90	0.94
PFSS total score		16.29	9.39	0.92	0.95

**Note.** α = Internal Consistency; ICC = Intraclass Correlation Coefficient

**Fig. 1:**
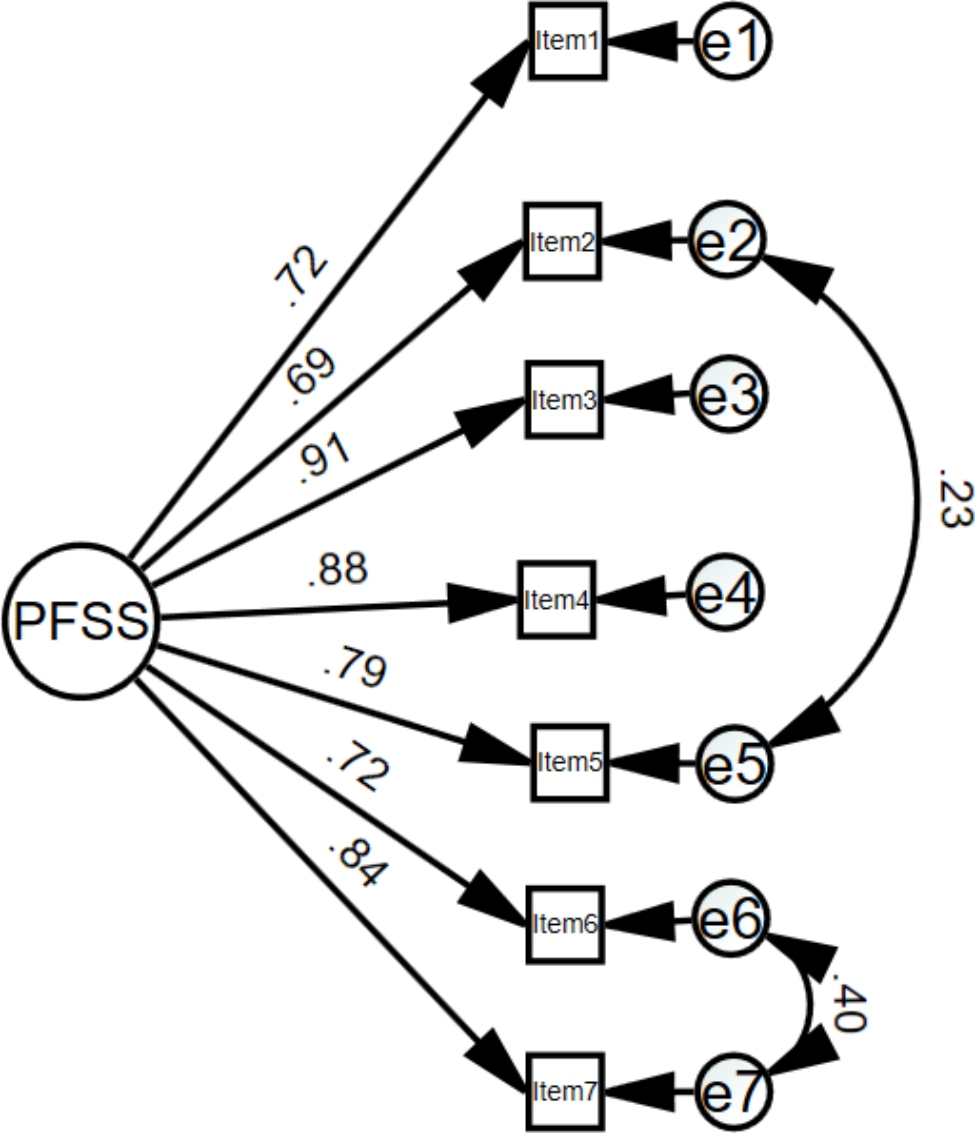
Confirmatory factor analysis of the P-PFSS. P-PFSS: Persian version of the Psychological Flexibility in Sport Scale

A one-factor structure solution of the PFSS was specified for the CFA. The one-factor solution for the P-PFSS was close to but did not meet adequate fit criteria (χ2 = 79.08, df = 14; CFI = 0.958; TLI = 0.937; RMSEA = 0.124; 90% CI = 0.098 − 0.152; SRMR = 0.035) [40, 41]. Whereas the CFI, TLI, and SRMR were acceptable, the RMSEA and χ2 were not. In order to improve the model, we checked the modification indices in order to improve the values of model fitness. Examination of the modification indices recommended that, allowing the error terms of item 6 (“Performance anxiety impairs my performance during competitions”) and 7 (“Worry makes my performance worse when I am competing”) as well as item 2 (“When competing I cannot control my nervousness, and my nervousness affects my performance negatively.”) and 5 (“It seems that most athletes can handle their feelings better than I do when they are competing.”) to covary and then re-estimated the model. Associations between these items are consistent with the conceptualization of sport sciences and psychological inflexibility; effects of loss of control (item 2 and item 5) and worry/anxiety (item 6 and item 7) on performance. Goodness-of-fit indices of modified one-factor model were χ2 = 23.2, df = 12; CFI = 0.993; TLI = 0.987; RMSEA = 0.056; 90% CI = 0.019 − 0.089; SRMR = 0.018 [40, 41]. The modified model of the Persian version of the PFSS fitted the data well (Fig. 1)

The convergent validity result showed adequate value. The AVE was 0.66, exceeding recommended values. Moreover, the results revealed that the low PF correlated with psychological health problems (depression, anxiety, and stress) and lower levels of quality of life (Table 4).

### Criterion validity

The criterion validity of the P-PFSS was assessed by examining the concurrent validity and the predictive validity. The correlational analysis between the P-PFSS and the AAQ-II was used to determine concurrent validity. As hypothesized, the P-PFSS scale presented correlation with the AAQ-II that evaluated similar constructs. The result revealed that the P-PFSS correlated with the AAQ-II (see Table 4).

**Table 4:** Pearson correlations between the P-PFSS and other scales

	P-PFSS	Depression	Anxiety	Stress	SWLS	AAQ
P-PFSS ^a^	–					
DASS-21-D ^b^	0.462**	–				
DASS-21-A ^c^	0.422**	0.755**	–			
DASS-21-S ^d^	0.521**	0.885**	0.793**	–		
SWLS ^e^	− 0.486**	− 0.589**	− 0.358**	− 0.552**	–	
AAQ ^f^	0.587**	0.628**	0.564**	0.683**	− 0.513**	–

*Note*. ^a^ Persian Version of Psychological Flexibility in Sport Scale; ^b^ Depression, Anxiety and Stress Scale 21-Depression; ^c^ Depression, Anxiety and Stress Scale 21-Anxiety; ^d^ Depression, Anxiety and Stress Scale 21-Stress; ^e^ Satisfaction with Life Scale; ^f^ Acceptance and Action Questionnaire

** p < 0.01

Table 5 presents the correlations between the P-PFSS and athletic-related variables in order to test the predictive validity. All correlations between the P-PFSS total score and athletic-related items were significant. We found higher athletic-related scores in participants with better PF.

**Table 5:** The results of Pearson correlations between the P-PFSS and athletic-related items

Variable	1	2	3	4	5	6
1. PFSS	–					
2. Physical preparation before competition	− 0.341**	–				
3. Mental preparation before competition	− 0.501**	0.651**	–			
4. Performance in competition	− 0.342**	0.801**	0.600**	–		
5. Physical recovery after competition	− 0.182**	0.383**	0.273**	0.329**	–	
6. Mental recovery after competition	− 0.286**	0.421**	0.429**	0.363**	0.610**	–

* p < 0.05; **p < 0.01

### Known-groups validity

Table 6 presents the results of the Student’s t test and ANOVA to determine known-groups validity. The P-PFSS mean score was significantly higher in older athletes (< 20 years old) and athletes with bachelor educational degree, but the differences in other background variables were not statistically significant.

**Table 6:** Differences in the P-PFSS total score between known-groups

Variable	M	SD	F/t
Age			-4.01**
Younger (≥ 20)	14.39	8.5	
Older (< 20)	18.66	9.2	
Gender			-0.44
Female	16.07	9.08	
Male	15.59	9.06	
Marital status			2.43
Single	15.35	8.9	
Married	17.5	9.04	
In a relationship	19.19	9.2	
Education			9.67 **
High school	13.85	8.2	
Diploma	17.59	8.3	
Bachelor	20.83	9.2	
Master and higher	15	8.8	
Economic status			1.4
Lower-middle and middle	16.46	8.9	
Upper middle	14.95	9.2	
Competition level			1.42
Beginner	15.73	8.5	
Club or university	15.39	7.2	
Provincial	15.13	8.9	
National	18.39	11.2	
International	14.95	8.6	
Sport type			-1.49
Individual	15.08	8.2	
Team	16.65	9.8	

* p < 0.05; **p < 0.01

### Reliability

The global Cronbach’s alpha was 0.92, and Cronbach’s alpha coefficients for the item-by-item reliability analysis ranged from 0.905 to 0.922. The CR was 0.92 and adequate. To determine the test-retest reliability of the P-PFSS, a sub-sample of athletes were asked to complete the instrument again after four weeks (N = 37). The mean score was 16.1 (SD = 7.33) at the test and 16.4 (SD = 7.4) at the retest. The Intraclass Correlations (ICC) was 0.95 for the total score (95% Confidence Interval, CI: 0.92–0.97, *p* < 0.00) and ranged from 0.85 to 0.97 for each item. Results indicated high test-retest reliability for the Persian version of the PFSS.

## Discussion

The purpose of the current study was to adapt the PFSS to Persian culture and language and evaluate the scale’s psychometric properties for Persian speaking athletes. Results revealed that the P-PFSS has a satisfactory validity and reliability. Also, the P-PFSS exhibited acceptable internal consistency and satisfactory test-retest reliability in the evaluated sample.

Results showed that the one-factor solution explained 69.65% of the total variance, and the CFA of the Persian version of PFSS confirmed the scale’s unidimensionality. Our results are in line with the findings of Johles et al., 2020, who explored a unidimensional factor that emerged with all seven items measuring psychological flexibility in athletes using CFA procedures [9]. However, the fit indices differed in the current study compared with the Swedish sample. The one-factor model with modifications was tested to examine the factor structure. Four items covaried via latent factors (i.e., item 2 and item 5, and item 6 and item 7). The modified model had a better fit within the Iranian culture context [40, 41]. Previous studies showed that anxiety/ worry and sports performance are related and higher levels of anxiety negatively affects sports performance [45, 46]. Moreover, loss of control in the form of lack of coping strategies and confidence has been found to influence performance negatively [47, 48]. It seems that the negative impact of loss of control and worry/anxiety are more prominent in Iranian athletes compared with Swedish athletes. One possible explanation for these findings could be that Iranian sports policy does not seem to be sufficient to support athletes such as providing adequate funding support, access to jobs, and updated knowledge. Consequently, Iranian athletes are likely to be highly concerned about their performance compared to Swedish athletes. Another possible explanation of these results is that athletes in Iran may not see sports as an opportunity to shape their future because of economic and political conditions. Therefore, the role of cultural, economic, social, and political factors needs to be considered in studying psychological flexibility.

The P-PFSS score was positively correlated with psychological health problems and negatively related to quality of life. These findings are in line with previous studies showing that PF is associated with a wide range of psychopathology dimensions, such as symptoms of anxiety, stress, depression, and sleep difficulty [49–52] and the same results have been found in the contexts of sport [9, 53–55]. We also found negative relationships between the P-PFSS and quality of life. This result is consistent with previous studies indicating strong associations between PF and positive psychological outcomes such as quality of life and acceptance and action [56–58].

Furthermore, the P-PFSS showed good criterion related validity. The P-PFSS was positively correlated with the AAQ-II and negatively correlated to all athletic-related variables, including physical and mental preparation before competition, performance in competition, and physical and mental recovery after competition. It could be concluded that both the P-PFSS and the AAQ-II measured a similar construct. It can also be assumed that PF is associated with the athlete’s sport-related behaviors. These findings are in line with previous studies showing that PF is related to preparation before competition [59], sport performance [6–9], and rehabilitation process [60] through accepting unpleasant thoughts and emotions.

The P-PFSS displayed sufficient ability to differentiate between known groups in the present study by observing the differences in the P-PFSS total score as a function of age and education. A previous meta-analysis of 46 studies found that older adults showed greater psychological flexibility compared to younger adults [61]. The results were inconsistent with our findings demonstrating that PF was significantly greater in younger athletes. One possible explanation for these findings could be the decreasing capacity of physical performance and the increasing risk for developing chronic diseases in older athletes compared to younger athletes [62–64]. Another possible explanation of these results is the timing of data collection. Data for the current study was collected during the COVID-19 pandemic. COVID-19 has created additional challenges for athletes by changing most aspects of athletes’ lives, with concerns about recovery from COVID-19, disruption of athletic training and competition, and worries about returning to sports [65–67]. Therefore, it is reasonable to conclude that older athletes have been the most affected by COVID-19 and consequently have less PF compared with younger athletes. Lastly, another possible explanation is that older people generally could be less likely to engage in sport and instead pursue other leisure activities. Furthermore, the results revealed that PF was significantly lower among athletes who had a bachelor educational degree.

The P-PFSS showed excellent reliability. The results indicated that the 7-item scale of the P-PFSS has high internal consistency (α = 0.92; CR = 0.92). Furthermore, a four-week test-retest revealed acceptable test-retest reliability for the scale (ICC = 0.95).

### Implication

The P-PFSS will enable the measurement of PF in Persian speaking athletes, both as an outcome and a process measure. From a theoretical standpoint, it opens the opportunity to further explore PFs role in athletic performance, mental health, rehabilitation of sport injuries etc. Since the P-PFSS is a general sport measure, it could be used in different athletic contexts and could be a source of inspiration for further sport specific scale developments. Further, PF is a key psychological construct targeted in numerous psychological interventions in sport psychology, and the P-PFSS therefore has the potential to facilitate future research of psychological interventions applied with Persian speaking athletes.

### Limitations and strengths

Before concluding, some limitations of the study should be addressed. First, the timing of data collection may have had an impact on the findings. More specifically, the data for this study was collected during the different peaks of the COVID-19 pandemic in Iran. Second, all the data in the current study were cross-sectional, meaning that direction of causation cannot be determined. Third, we used a convenient sampling method in this study that limit the generalizability of findings. Fourth, the PFSS measures PF as the overarching construct. As the construct of psychological flexibility is multifaceted, there is also a need to develop more instruments on a sport specific level. One suggestion is that researchers use the P-PFSS and the PFSS as an inspiration in the development phase and use both scales in trials to be able to compare results and further explore the validity of the instrument. Finally, dimensions of life satisfaction (e.g., psychological, environmental, physical, and social) were not considered in the current study, which did not enable us to ascertain which dimensions of life satisfaction might be more affected by the PF.

On the other hand, this study has several strengths, which include an adequate sample size, a wide range of ages, a broad range of athletes from different sports, and an investigation conducted in another cultural context than Swedish-speaking athletes.

## Conclusion and future directions

In conclusion, the current study outlines that the Persian version of the PFSS has satisfactory psychometric properties similar to those of the original Swedish version. The findings provide superior psychometric properties for the PFSS, including validity and reliability for assessing context-specific measures of PF in the athletic populations.

Future research can use this scale as an outcome or mediator measure of ACT and MA programs in the context of sport. The sensitivity of the scale needs to be tested in longitudinal intervention trials. Also, future research needs to evaluate the dynamic changes in psychological flexibility in athletes using longitudinal studies. There is also a need to develop more instruments on a sport specific level and one suggestion is that researchers use the P-PFSS and the PFSS as an inspiration in the development phase and in the actual trial uses both scales to be able to compare results and further explore the validity of the instrument. Moreover, information on cultural adaptions in studying psychological flexibility is still limited. Therefore, the impact of culture on PF needs further exploration.

## Data Availability

The datasets used and/or analyzed during the current study are available in the Open Science Framework, [https://osf.io/wbqn3/**].**
